# Enhanced Sensitivity in Neurotoxicity Detection via Cross-Correlation of Spectroscopical and Electrophysiological Features

**DOI:** 10.1021/acsomega.5c04069

**Published:** 2026-06-24

**Authors:** Christian Tentellino, Marta d’Amora, Rustamzhon Melikov, Francesco Tantussi, Michele Dipalo, Francesco De Angelis

**Affiliations:** † Plasmon Nanotechnologies, 121451 Istituto Italiano di Tecnologia, Via Morego 30, 16163 Genoa, Italy; ‡ Department of Biology, University of Pisa, S.S. 12 Abetone e Brennero, 4, 56127 Pisa, Italy

## Abstract

Neurotoxicity detection using electrophysiological readouts is limited in sensing chemically induced variations across neuronal ion channels. Thus, supportive knowledge of the neuronal biochemical environment may unveil hidden neurotoxicity mechanisms. Herein, we integrated a spectroscopical, via Raman spectroscopy, and an electrophysiological, via microelectrode array, readout for monitoring the neurotoxic effects of gabazine, a known epileptiform inducer, toward neuronal rat primary cells. The monitoring and correlation of the spectroscopic and electrophysiological features, prior to and following the gabazine exposure, highlighted the advantages in the monitoring and correlation of these biological complementary features, suggesting an innovative and more sensitive approach for neurotoxicity detection in the drug discovery and development pipeline.

## Graphical abstract

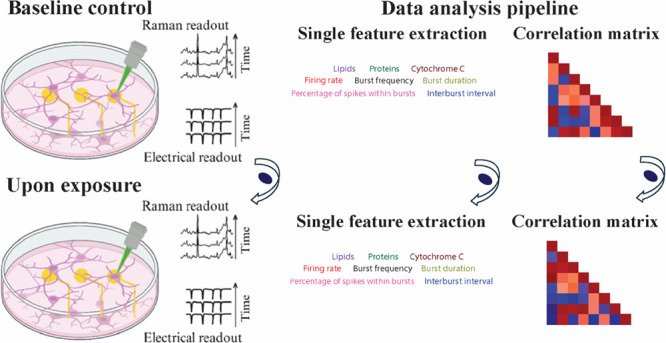

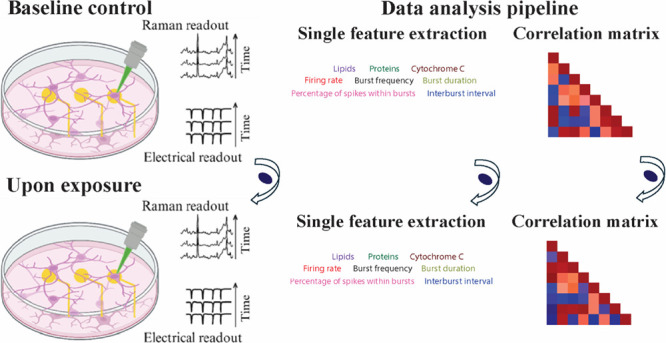

## Introduction

The drug discovery and development pipeline is a lengthy process, taking an average of 15 years to complete and costing around two billion dollars.^1^ It consists of different stages, moving progressively from the in silico drug design to preclinical and clinical trials, reviews and approval, and finally, to the launch of the drug in the market. Once in the market, any potential side effects of the drug are monitored and reported through a process known as pharmaceutical surveillance. Therefore, drug failures and withdrawals can occur prior to and following the launch in the market, respectively. Safety-related attrition plays a key role in halting novel drug-like candidates for cytotoxicity, representing the real bottleneck of the process. Neurotoxicity accounts for halting a quarter of the overall novel drug-like candidates, and it is commonly observed within the “late” drug discovery and development pipeline (clinical trials).^2^ Thus, it represents an economical burden and a danger to the health of human volunteers. If this is not alarming enough, several drugs in the market are under ‘tight’ prescription and monitoring due to adverse side effects on the neurological system, including suicidal intentions, sedation, abuse liability, and seizure/convulsion. In some cases, these side effects have led to drug withdrawal due to the small therapeutic window.^3^ All of these urge the design of novel tools to assess neurotoxicity (i) in the early stages of the drug discovery and development pipeline and (ii) with greater sensitivity, unveiling “hidden” neurotoxicity mechanisms responsible for the withdrawn of drugs during the pharmaceutical surveillance.

Functional neurotoxicity screening commonly exploits electrophysiological techniques such as patch clamp,^4^ voltage-sensitive dyes,^5^ and microelectrode array (MEA).^6^ While voltage-sensitive dyes and patch clamp technologies are perturbative to the biochemical environment and lead to end point analysis, due to the use of a micropipette encapsulating a recording electrode and fluorophores, MEA technologies offer a nondestructive readout of the electrophysiological activity of neuronal cells through biofeatures such as firing rate, burst frequency, interbursts interval, and mean burst duration.^7^ The advantages in using the MEA technology for a noninvasive and longitudinal recording were exemplified by Iachetta et al., who were able to monitor, by an end-point-free approach, the chronic pentamidine- and doxorubicin-induced cytotoxicity for longer than one month.^8^ Furthermore, MEA-based platforms land themselves for the recording of intracellular action potentials through a biocompatible phenomenon known as optoporation, which explores NIR-light pulses to monitor the intracellular action potential dynamics with greater sensitivity.^9^ This was exemplified by Melikov et al., who were able to capture distinctive and key subthreshold signals for assessing the properties of the neuronal network.^10^

Unfortunately, the MEA technology lacks in the identification of neurotoxicity mechanisms, which do not act toward ion channel receptors, including morphological and biochemical changes, which commonly reflect drug-induced toxicity. Furthermore, the net electrophysiological activity at the electrode may result null if simultaneous spikes counteract each other in phase and form, affecting the electrophysiological analysis over time. Phase and form of a spike depend upon the neuronal attachment and the neuronal type (serotonergic, GABAergic, etc.).^11^ Thus, improvements in neurotoxicity detection can be achieved by providing (i) an integrative, multiplexing, and nondestructive readout, (ii) real-time neuronal identification using spike-sorting analysis detection, and (iii) alternative approaches. With that purpose, Hartmann et al. reported a single spike-sorting analysis procedure for neuronal identification, resulting in the identification of single neurons via spikes’ phase and waveform and the monitoring of the single neuronal activity prior to and following chemical exposure.^12^ Meanwhile, Li et al. identified an ethylenic spectroscopical vibration associated with a genetically encoded rhodopsin-based voltage indicator, which mirrors subcellular local changes in membrane potential and lays the foundation for an alternative approach in neurotoxicity screening via hyperspectral stimulated Raman scattering microscopy.^13^ On the other hand, in our previous work, we have successfully integrated an electrophysiological readout, via MEA devices, with Raman spectroscopy, a nondestructive optical readout that provides an integrated and different perspective on the metabolic and health status at the single-cell level, including the possibility of sensing any potential neurotoxicity mechanism that acts on the de novo synthesis of biomolecules and ROS formation.^14^ We exemplified the advantages of integrating these two readouts, suggesting a novel, longitudinal, label-free, and real-time mean for monitoring the electrophysiological activity of neuronal rat cells.^14^ Specifically, this was achieved by following the spatiotemporal Raman scattering profile of cytochrome C.^14^ However, the potential of this integrated technology for neurotoxicity detection remained unexplored.

Leveraging previously reported results, herein we show an optimized protocol for the simultaneous extraction of the spectroscopic and electrophysiological activity prior to and following chemical exposure. We specifically exposed neuronal rat primary cells to gabazine, an epileptiform inducer, and monitored the correlation of the biofeatures prior to and following chemical treatment. Our study suggests that the correlation of the monitored biofeatures, namely, cytochrome C, lipids, proteins, spike frequency, burst frequency, interburst interval, percentage of spikes within a burst, and burst duration, provides a more sensitive readout than the independent MEA and Raman readouts (via the Mann–Whitney test), highlighting the greater sensitivity in the cross-correlation of features originating from an integrated spectroscopical–electrical readout.

## Experimental Section

### Cell Culture and Media Compositions

Primary rat hippocampal neurons were purchased from Lonza (R-HI-501, Lonza Walkersville, United States). According to a previously established protocol,^14^ neurobasal medium A (without phenol red) was supplemented with the B27 Supplement minus antioxidants (Thermo Fisher Scientific, Inc., Waltham, MA), l-glutamine (2 mM), gentamicin (50 μg/mL), amphotericin (37 ng/mL), and Neural Serum Factor 1 (NSF1, 2%) (from the Lonza PNGM Singlequots Growth Supplements) and used for cell culture.

Following the sterilization of the chips occurred within the cell culture hood and by exposure to UV light for 30 min, the culture surface area of the MEAs was coated with a solution of poly-d-lysine (30 μg/mL, Sigma-Aldrich, St. Louis, MO, USA) and laminin (2 μg/mL, Sigma-Aldrich, St. Louis, MO, USA) in phosphate-buffered saline (PBS, pH = 7.4, Thermo Fisher Scientific, Inc., Waltham, MA) for 1 h at room temperature, to increase the cell adhesion on the chips. Thus, the MEAs were rinsed three times with sterile water (Sigma-Aldrich, St. Louis, MO, USA) and dried inside the hood, before cell seeding. Primary rat hippocampal neurons were seeded on the devices and incubated (37 °C, 5% CO_2_, 95% humidity) for 4 h. Following adhesion, a portion of the medium was gently removed, and a fresh and prewarmed medium was introduced. The cell culture was sustained for 3 weeks. On day 5, half of the medium was replaced with a fresh and prewarmed medium. The same procedure was routinely carried out every 3–4 day until the spectroscopical and electrophysiological measurements.

### Raman Spectroscopy

A Renishaw InVia was used for Raman imaging. The samples were imaged using a 532 nm excitation wavelength, a laser power of 13 mW at the objective, and a 60× water-immersion lens objective (NA = 1). The peak center was set at 1200 cm^–1^, with an integration time of 0.4 s, 1 accumulation, a step size of 3 μm, and high-confocality mode. The size of the Raman maps collected in the simultaneous electrophysiological–spectroscopic analysis was 30 × 30 μm^2^. The required time for recording a 30 × 30 μm^2^ Raman map is 2.5 min, which corresponds to a total of 12.5 min of Raman acquisition per condition. Raman thermostable measurements were carried out using out-of-focus imaging and a chamber designed for “live-cell biocompatible” measurement conditions (37 °C and 5% CO_2_). This minimized the Raman spectra fluctuations caused by temperature changes at the focal point. We invite the readers to check our previous publication for more details.^14^

In contrast to our previous work, which focused on the longitudinal recording of single cells,^14^ herein we use Raman spectroscopy to characterize biochemical events occurring at the single-cell level across the whole neuronal cell culture following treatment with gabazine (30 μM, 10 min). We opted for this approach to capture the phenotypical heterogeneity across the whole neuronal cell culture with higher throughput than classical spontaneous Raman spectroscopy, while eliminating interference from temporal dynamics and different cellular shapes during data analysis and interpretation. For sake of clarity, the Raman spectra collected from a 30 μM and a 1 mM gabazine solution, using the aforementioned acquisition settings, was negligible and comparable to stochastic photon fluctuations (data not shown).

### Data Processing

Raman data were collected and processed in two stages. Preprocessing was performed using WiRE 5.5 and included the following steps: truncation, cosmic ray removal, noise filtering, baseline subtraction, and smoothing (Figure S1, panel 2). The truncation was carried out maintaining the Raman spectrum from 500 to 1720 cm^–1^. The cosmic ray removal was carried out in two steps: (i) using a detection method with the width parameter and height parameter set as 3 and 15 and (ii) using the nearest neighbor algorithm with the noise level set as 0.89 and the scaling factor 10 as well as the spectrum height set as 6.63 and the scaling factor 50%. The noise filter was based upon a WiRE 5.5 algorithm of principal component analysis, which results in the generation of a software interface in which the components (expressed as loadings) can be manually selected to discriminate the noise from the Raman signals. Baseline subtraction was performed using a polynomial order of 12 and a noise tolerance of 1.5. Finally, the Raman spectra were smoothed using a Savitski–Golay filter with a smoothing window of 9 and a polynomial order of 3.

Then, the preprocessed data were imported in MATLAB where the data were further processed. The MATLAB script used was written implementing an existing one.^14,15^ The preprocessed dataset was normalized to the Raman scattering intensity of phenylalanine at about 1004 cm^–1^ and then used to calculate the median corresponding to each Raman map, prior to and following gabazine exposure (Figure S1, panel 2). The accuracy of calculating the median Raman spectrum for each map therefore depended on both the acquisition step size and the size of the designed area. Then, the five medians per sample associated with each condition, prior to and following gabazine exposure (30 μM, 10 min), were averaged to generate a representative Raman spectrum of the sample prior to and following the chemical treatment (Figure S1, panel 3). We carried out this procedure for nine independent samples over four different neuronal cultures (Figure S1, panel 4), resulting in the collection and analysis of a total of 10890 Raman spectra, 5445 for each condition. The whole data analysis pipeline schematic is shown in Figure S1.

The overall data processing and analysis do not exploit the use of operator-defined masks to extrapolate the Raman spectra at any step. Accordingly, the data processing and analysis pipeline is fully automated and free of any interference from the operator. This approach is also based upon equal contribution from each sample and condition, avoiding misleading outcomes due to an unbalanced dataset.

### Electrophysiological Measurements

Electrophysiological measurements were carried out using a MEA2100 (MEA2100-LITE-System) utilizing TiN MEAs with an electrode diameter of 30 μm and a spacing of 200 μm. High-pass and low-pass filters were set at 100 Hz (2nd order) and 3500 Hz (4th order), while the sample rate was set at 25 kHz. The neuronal activity considering the whole active electrodes was calculated through the Multi Channel Analyzer and included the following variables: firing rate, burst frequency, burst interval, burst duration, and percentage of spikes within bursts.

In the standard approach, spikes were detected using a global threshold set at five times the standard deviation of the background noise, applied uniformly across all active electrodes. However, in some recordings, spike amplitudes and noise levels varied significantly between electrodes, resulting in a suboptimal global threshold. In these specific cases, we applied individual thresholds to each active electrode based upon the specific signal-to-noise ratio. To ensure consistency and reproducibility, these electrode-specific thresholds were determined prior to drug exposure and then maintained for the post-treatment analysis. No adaptive or dynamic rethresholding was applied after drug application. This approach ensured that any changes observed in spike activity could be attributed to the pharmacological treatment rather than to alterations in detection parameters. The electrophysiological recording was carried out for ca. 10 min prior to the gabazine exposure. Then, the neuronal cell culture was treated with the epileptic inducer form gabazine (30 μM for 10 min) or the vehicle control (DMSO, 0.1% for 10 min). Following the drug treatment, the electrophysiological neuronal activity was recorded for 10 min and compared to the baseline activity.

Prior to any electrophysiological baseline recording, the neuronal cell culture was allowed to equilibrate in the chamber for “live-cell biocompatible” measurement conditions for ca. 15 min, which in our case was the time needed for the neuronal cell culture to restore its electrophysiological activity. The customized stage incubator was used to minimize longitudinal changes across the neuronal cell culture, namely, the pH and the buffering capacity of the biological medium, the temperature and the humidity at the sample, as well as the osmolarity of the biological medium. The stability of the neuronal electrical recording over the full recording period in the absence of chemical perturbation was demonstrated in our previous publication.^14^

### Additional Details on the Synchronous Recording of Electrical and Spectroscopical Features

Regarding the synchronous collection of spectroscopical and electrophysiological data, the overall collection of five Raman maps required a slightly longer time (12.5 min) than the actual electrophysiological readout (10 min), resulting in the creation of small-time windows in which only Raman imaging was carried out. Notably, the synchronous collection of spectroscopic and electrophysiological features following the drug treatment occurred without any washing attempt. An aliquot of the biological medium was used to dilute gabazine to the working concentration and was directly poured back into the sample.

### Biocompatible Recording Conditions

Special attention has been given to maintaining the physiological conditions of the neuronal cell culture during the whole experiment. While the temperature (set at 37 °C) was managed directly by the Multi Channel System MCS GmbH, other parameters, such as CO_2_ and humidity, required the development of a custom chamber to be integrated between the Raman microscope and the Multi Channel System MCS GmbH. This home-built chamber allowed the entrance of the observation objective from the top, and from the side wall, the flow of a heated and humidified 5% CO_2_–air mixture (provided by an Okolab system; Okolab Stage Top Digital Gas (oko-lab.com). The small losses of the chamber were compensated by the low incoming gas flow, ensuring that a suitable condition for long-term recording was reliably established.

### Data Analysis

Single feature analysis was carried out using the nonparametric Mann–Whitney test. The significance level was set to 0.05. The cross-correlation analysis was carried out using the Pearson correlation app in OriginPro 2025 (correlation plot), and the significance level was set at 0.05.

## Results and Discussion

### Development of a Protocol for the Investigation of Neurotoxicity

In our previous work, we have developed an integrated spectroscopical–electrophysiological platform for in-depth longitudinal biological characterization of primary neuronal rat cells.^14^ Specifically, we optimized a protocol for the longitudinal recording of the vibrational–electrophysiological activity considering the single neuronal cells for the Raman measurements and the whole cell culture for the electrophysiological measurements. However, as single neuronal cells may respond differently to the chemical exposure in relation to their metabolic state, herein, we opted to design a novel protocol that better suits the screening of neurotoxicity of exogenous chemicals. As the Raman and the electrophysiological readouts do not cross-talk or interfere with each other,^14^ we opted to simultaneously carry out Raman imaging and electrophysiological recording prior to and following the gabazine exposure (30 μM, 10 min). The drug concentration and incubation time were chosen based on the previously established neurotoxicity studies from Zhai et al. and Annika et al.^16,17^ Five Raman maps were collected during the recording of the electrophysiological activity of neuronal rat cells. Raman mapping was carried out according to a previously established protocol.^14^ With the purpose of correlating the spectroscopical and electrical features prior to or following the chemical exposure, the five medians measured per each condition were averaged (Figure S1, panel 3) and associated with the respective electrophysiological activity of the neuronal rat primary cells prior to or following the gabazine exposure. The key points of the methodology used in this experiment are shown in Figure 1.

**Figure 1:**
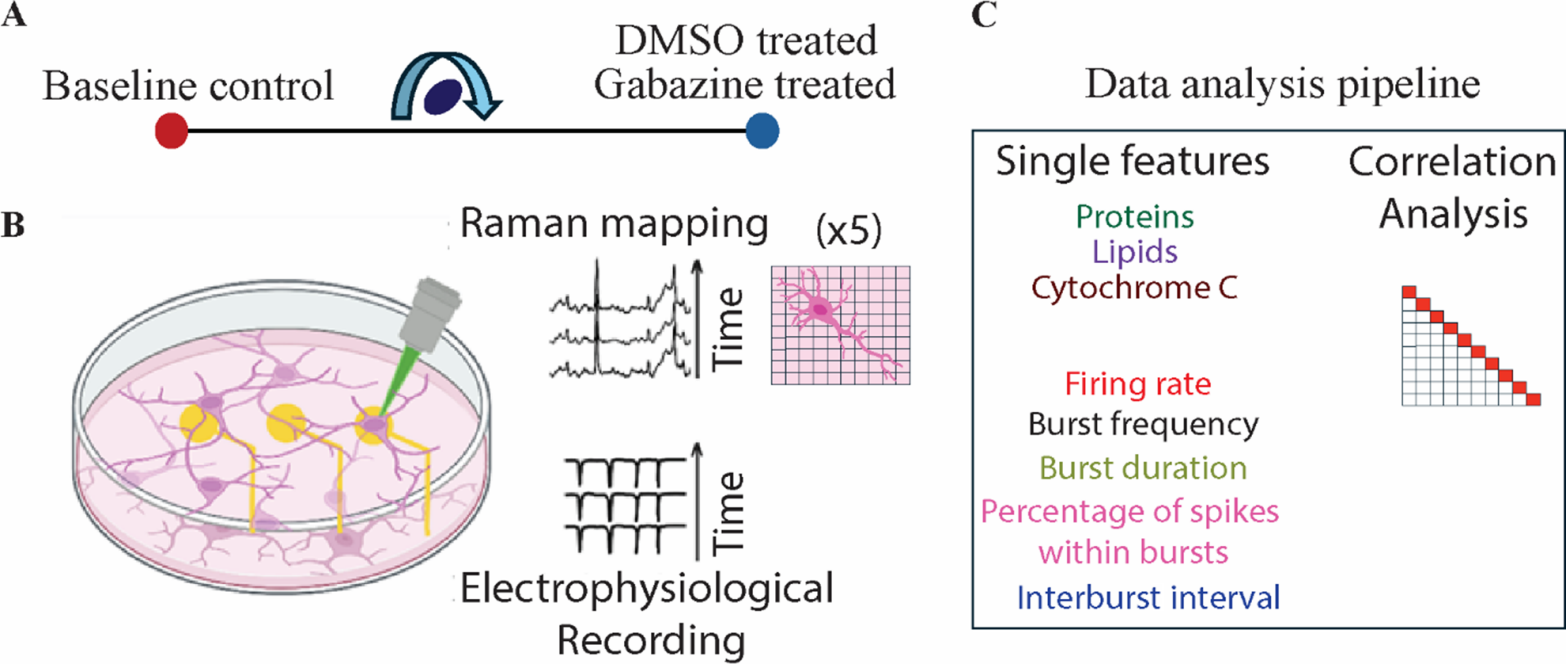
Illustration of the experimental procedure used in this work. (A) Spectroscopical and electrophysiological activities of neuronal rat cells were recorded to establish a baseline activity level (red dot) prior to the drug treatment, followed by further electrophysiological and spectroscopical recordings (blue dot). As proof of principle, primary neuronal rat cells were treated with the inducer of acute epileptic form, gabazine (30 μM, 10 min). (B) Experimental procedure used for the simultaneous recording of electrophysiological (10 min) and spectroscopical features (5 Raman maps) associated with the neuronal cell culture prior to and following exposure to either gabazine (30 μM, 10 min) or DMSO (0.1%, 10 min). (C) Data analysis pipeline used in this work. Initially, the comparison of single spectroscopical and electrophysiological features prior to and following the gabazine treatment was carried out. After that, the data analysis pipeline takes into account the cross-correlation of the electrophysiological and spectroscopical features of the whole data set.

### Cross-Correlation of Features as a Novel Approach for Neurotoxicity Screening

Gabazine exposure is known to induce acute epileptic forms that are mediated by a higher firing rate and a change in the burst profile.^15,16^ This is clearly observed in our seven paired samples originating from two different neuronal cultures before and after the chemical exposure. The neuronal electrophysiological activity was recorded in the form of extracellular spikes (Figure S2A1,A2) and exhibits the typical negative phase shape of extracellular field potentials (Figure S2B1,B2). Consistent with our previous findings,^14^ Figure S2A1,A2 illustrate a stable neuronal electrophysiological activity throughout the 10 min of recording period, unaffected by the continuous laser exposure required for Raman imaging.

However, as highlighted in the work of Hartmann et al.,^12^ different neuronal types and attachment over the electrodes may lead to changes in the detected number of spikes per time, a null electrophysiological activity on the electrodes, and a different signal-to-noise ratio. Due to these reasons, the electrophysiological readout is commonly characterized by a high variance across the data set and often results in a challenging data analysis. Therefore, for the sake of standardization and consistency, our data analysis and feature extraction exploited a commercially available platform, the Multi Channel Analyzer, and the default spike detection and analysis options. Overall, the detection of the single spikes was based upon the negative phase of the neuronal spike and was carried out by using a threshold value of five times the standard deviation of the background noise level. The only exception to the methodology occurred when neuronal spikes were characterized by different amplitudes across the active electrodes. In such a case, we applied a specific threshold associated with a specific electrode, depending upon the signal-to-noise ratio. Worthy of note, the specific applied threshold was maintained prior to and following gabazine exposure to guarantee consistency in the extraction and analysis of the electrophysiological data. The extracted and investigated features were firing rate, burst frequency, burst interval, burst duration, and percentage of spikes within bursts.

The electrophysiological analysis of the whole data set prior to and following the exposure to gabazine was observed to be nonsignificant using a Mann–Whitney test, a classical approach for an asymmetric distributed dataset. Worthy of note, only the changes in the neuronal firing rate prior to and following the exposure were close to being significant, with a *p* value of 0.05528 when a Mann–Whitney test was carried out, with the rest of the electrophysiological features being far away from statistically significant differences prior to and following the gabazine exposure (Figure 2E). For the sake of clarity, the *p* values of the Mann–Whitney test for the burst frequency, interburst interval, burst duration, and percentage of spikes within the burst were 0.70148, 0.60928, 0.0967, and 0.20134, respectively (Figure 2A–D). For the sake of clarity, we also measured the electrophysiological activity of two neuronal cell cultures prior to and following the treatment with the vehicle control (0,1% DMSO for 10 min), which was not expected to produce any effect (Figure S3A–E). Across these two neuronal cell cultures, the electrophysiological activity showed fluctuations following the treatment with the vehicle control, suggesting that an integrated approach could ameliorate the reliability of the electrophysiological measurements for neurotoxicity detection. We therefore proceed to the analysis of the spectroscopical data. The average Raman spectrum associated with the primary neuronal rat cells is shown in Figure 2F, while the Raman scattering associated with the cytochrome C, lipids, and proteins is shown in Figure 2G.

**Figure 2:**
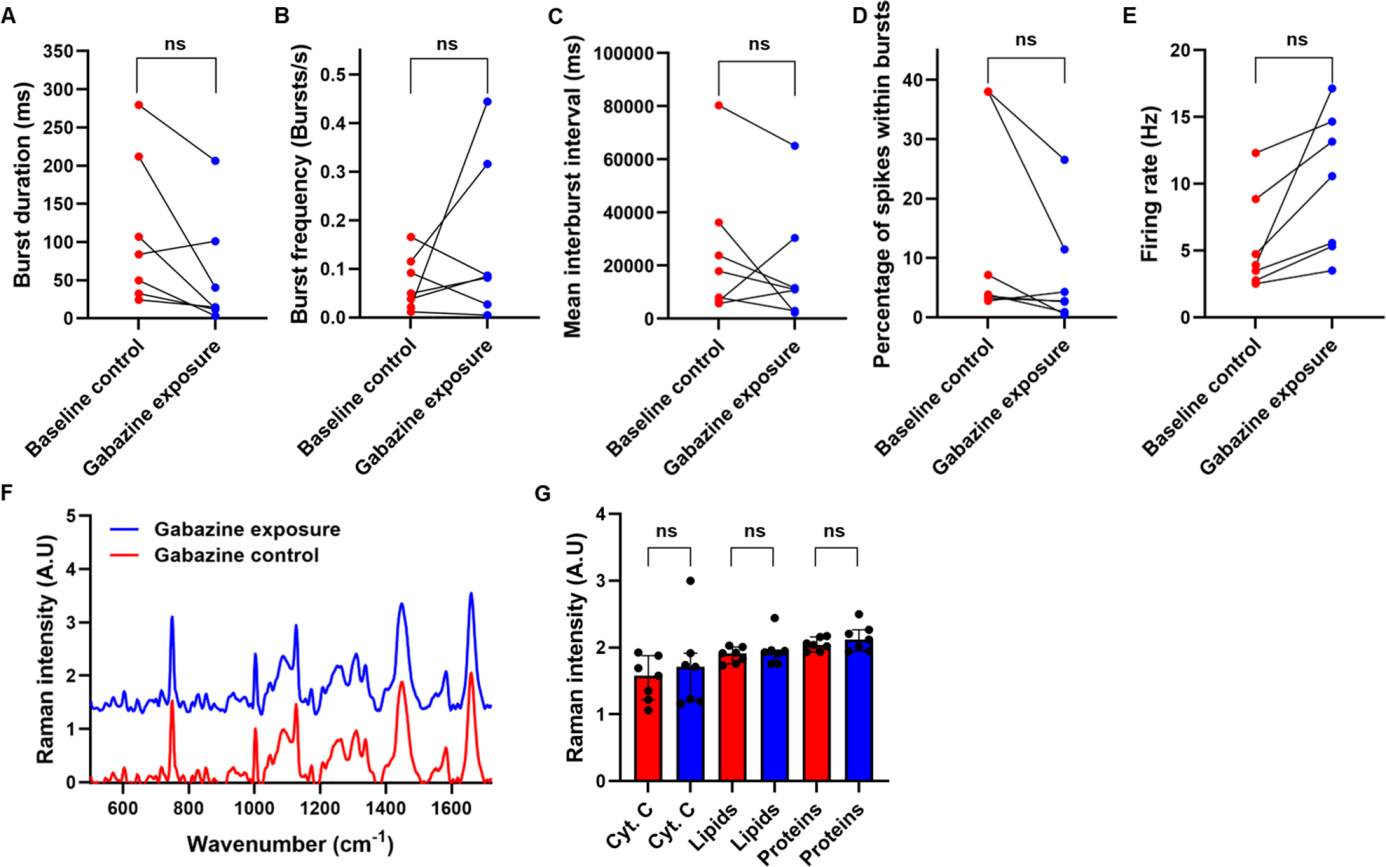
Nonstatistical significance difference between the electrophysiological and vibrational features of primary neuronal rat cells prior to and following the exposure to gabazine (30 μM, 10 min) and a single feature Mann–Whitney analysis. (A) Changes across the burst duration in primary neuronal rat cells prior to and following chemical exposure. (B) Changes across the burst frequency in primary neuronal rat cells prior to and following chemical exposure. (C) Changes across the interburst interval in primary neuronal rat cells prior to and following chemical exposure. (D) Changes across the percentage of spikes within bursts in primary neuronal rat cells prior to and following chemical exposure. (E) Changes in the firing rate of primary neuronal rat cells prior to and following the exposure to gabazine (30 μM, 10 min). (F) Average Raman spectrum of primary neuronal rat cells measured from two different neuronal cultures and seven different samples prior to and following the gabazine exposure. (G) Relative Raman scattering of cytochrome C (750/1004) cm^–1^, lipids (1450/1004) cm^–1^, and proteins (1660/1004) cm^–1^ prior to (red) and following the gabazine exposure (blue). The data are illustrated as median (bar values) ± IQR. The single dots in each column represent the average relative intensity value calculated for each sample prior to and following gabazine exposure. The Mann–Whitney tests took into account two different neuronal cultures and seven different samples prior to and following the exposure to gabazine.

In accordance with the electrophysiological analysis, via single-feature analysis, the average Raman spectrum of primary neuronal rat cells, prior to and following the gabazine exposure, does not suggest any remarkable change upon exposure to the gabazine or vehicle control (Figures 3F and S3F).

**Figure 3:**
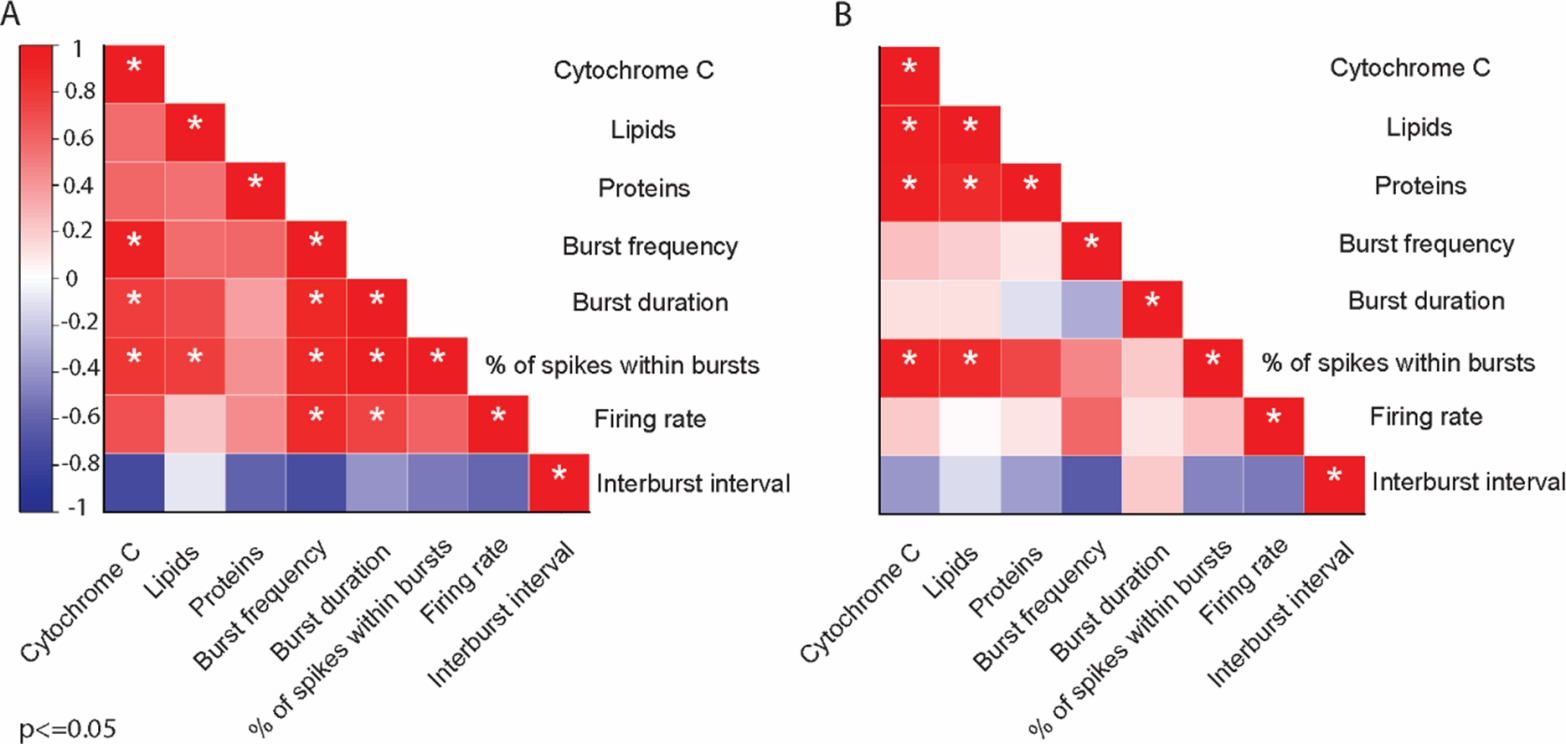
Pearson cross-correlation of the biofeatures measured by an integrated spectroscopical–electrophysiological readout. The Pearson cross-correlation of biofeatures (A) prior to and (B) following gabazine exposure (30 μM, 10 min). Statistical significance is shown with an * and is assigned to a Pearson correlation p-value ≤ 0.05. In red and blue are reported the positive and negative correlations, respectively. On the other hand, features that do not correlate tend toward white. The cross-correlation measurements were carried out over two different neuronal cultures and 7 different samples prior to and following exposure to the gabazine.

We therefore investigated more closely the Raman changes associated with the cytochrome C, lipids, and proteins that are known to promptly respond to cell stressors.^18–21^ Cytochrome C is a key player in the intracellular environment, modulating processes such as the ATP synthesis and actively participating in ROS scavenging and apoptosis.^18,22^ Notably, the rate in ATP synthesis and the neuronal action potential are strongly interlinked by the cytochrome C oxidase.^23^ Lipids are also important in the early neurotoxicity, cardiolipin peroxidation modulates the release of cytochrome C from the mitochondria to the cytoplasm,^24^ while lipid droplet accumulation occurs during oxidative stress and inflammation.^25^ Lastly, drug-induced changes in the relative abundance, distribution, and folding of proteins may be reflected by spatial and spectral changes of the Raman spectrum.^26^

Taking into account the highest Raman band associated with each biomolecule, a Mann–Whitney test was used to investigate whether the Raman changes observed upon gabazine exposure were statistically significant. Specifically, the Mann–Whitney test did not observe any significant change upon gabazine exposure when the relative Raman scattering of these biomolecules was compared prior to and following gabazine exposure, Namely, cytochrome C, lipids, and proteins with a *p* value of 0.7983, 0.7983, and 0.37109, respectively. For the sake of clarity, the single values for each feature, prior to and following the gabazine exposure, are shown in Table S1. Furthermore, the Mann–Whitney test values for each feature, prior to and following the gabazine exposure, are reported in Table S2. A stability over time was similarly observed prior to and following the administration of the vehicle control (0.1% DMSO, 10 min) (Figure S3G). Overall, these results suggest that an integrated and multiplexed analysis may represent a more powerful tool for data analysis and neurotoxicity screening upon neuronal rat cell exposure to chemicals. With the purpose of investigating this hypothesis, we carried out a large cross-correlation of the whole spectroscopical–electrophysiological features. Namely, firing rate, burst frequency, interburst interval, burst duration, percentage of spikes within bursts, lipids, proteins, and cytochrome C to observe any changes in the cross-correlation of the features prior to (Figure 3A) and following the gabazine exposure (Figure 3B) using the same data set achieved from a total of seven paired samples and two different neuronal cultures. For sake of clarity, the Pearson correlation values among the biofeatures prior to and following the gabazine exposure are reported in Figures S4 and S5, respectively.

Figure 3A,B shows the cross-correlation changes among the biofeatures prior to and following exposure of primary neuronal rat cells to gabazine (30 μM, 10 min), respectively. Alternatively, while analysis of the single features showed no significant changes prior to and following gabazine exposure, significant changes were observed in the cross-correlation of these variables, more likely reflecting an action toward the neuronal health state of gabazine and indicating greater sensitivity in monitoring the overall health state of a neuronal cell culture. Specifically, in Figure 3A, which represents the baseline control measurements, a statistical significance correlation was measured between cytochrome C and burst frequency, burst duration, and the percentage of spikes within bursts. Worthy of note, a Pearson value close to 0.7 was measured between cytochrome C and the firing rate, suggesting a strong coupling of these features even considering the metabolic heterogeneity of the neuronal cells within the neuronal monolayer. On the other hand, a weak correlation between cytochrome C, lipids, and proteins was observed (Pearson correlation value about 0.5). Significant Pearson correlations were also measured between lipids and the percentage of spikes within bursts; between the burst frequency and the burst duration, the percentage of spikes within bursts, and the firing rate; as well as between the burst duration, the percentage of spikes within bursts, and the firing rate.

Figure 3B shows the cross-correlation changes of biofeatures following the exposure of primary neuronal rat cells to gabazine. Quite significant changes in the cross-correlation of these features were observed with respect to the baseline control samples. The exposure to gabazine results in the loss of Pearson cross-correlation between cytochrome C, burst frequency, and burst duration, while new, positive, and significant cross-correlations were observed between cytochrome C, lipids, and proteins as well as between lipids and proteins, suggesting that these molecules may have established a tighter metabolic network upon gabazine treatment to sustain the increased electrical activity (see the firing rate). Conversely, gabazine exposure caused a loss in the significant correlation among the electrophysiological features, namely, burst frequency, burst duration, percentage of spikes within bursts, and the firing rate. Only the Pearson cross-correlations between the percentage of spikes within bursts and cytochrome C and lipids were stable following the exposure of the neuronal rat primary cells to gabazine.

On the basis of the results of our preliminary study, we speculate that the relationship between cytochrome C and the electrophysiological features is key to early sense neuronal cytotoxicity, and exploring these relationships may unveil biochemical mechanisms behind neurodegenerative diseases. Furthermore, our study suggests that an integrated spectroscopical–electrophysiological readout that enables simultaneous monitoring of multiple variables and cross-correlation of them leads to a greater sensitivity in the neurotoxicity detection of novel drug-like candidates and may contribute to a more efficient drug discovery and development pipeline. Our study implies the possibility of developing a novel approach to investigating and sensing the mechanism of neurotoxicity. Specifically, the cross-correlation matrices of toxic compounds with known mechanisms of action can be fed to a deep-learning model to identify and characterize the means through which yet-to-explore molecules exert, if any, neuronal side effects, thereby facilitating a large throughput screening of chemical libraries within the drug discovery and development pipeline. In conclusion, we propose this approach as a potential means for a more robust detection of neurotoxicity, with necessary further studies and validation, ideally conducted by independent research groups.

## Supplementary Material



## Notes

Additional material and methods, data processing pipeline, additional experimental data and additional references (PDF)

## 3

Morgridge Institute for Research, 330 N Orchard Street, Madison, WI 53715, USA
