# Supplementary material for: Enhanced Sensitivity in Neurotoxicity Detection via Cross-Correlation of Spectroscopical and Electrophysiological Features
Source: ACS Omega. 2026 Jun 24;11(26):38371–8. doi: 10.1021/acsomega.5c04069 (PMC13347391; doi:10.1021/acsomega.5c04069)
Supplement: Supplementary file 1 [file ao5c04069_si_001.pdf]

Enhanced sensitivity in neurotoxicity detection via cross-correlation of spectroscopical and electrophysiological features.

Electronic Supplementary information

Christian Tentellino,[1,3*] Marta d'Amora,[1,2] Rustamzhon Melikov,[1] Francesco Tantussi,[1] Michele Dipalo[1] and Francesco De Angelis[1]

[1]Istituto Italiano di Tecnologia, Department of Plasmon Nanotechnologies, Via Morego 30, Genoa, 16163, Italy

[2]University of Pisa, Department of Biology, S.S. 12 Abetone e Brennero, 4 , Pisa, 56127, Italy

[3]Current address: Morgridge Institute for Research, 330 N Orchard Street, Madison 53715, Wisconsin

Contents

Material and Methods……………………………………………………………………………2

Figure S1……………………………………………………………………………………………4

Figure S2……………………………………………………………………………………………5

Figure S3……………………………………………………………………………………………6

Table S1……………………………………………………………………………………………7

Table S2……………………………………………………………………………………………9

Figure S4……………………………………………………………………………………………9

Figure S5……………………………………………………………………………………………10

References…………………………………………………………………………………………11

Experimental section

Cell Culture and media compositions

Primary rat hippocampal neurons were purchased from Lonza (R-HI-501, Lonza Walkersville, United States). According to a previously established protocol,[1] neurobasal medium A (without phenol red) was supplemented with the B27 Supplement minus antioxidants (Thermo Fisher Scientific, Inc., Waltham, MA), L-glutamine (2 mM), gentamicin (50 µg/mL), amphotericin (37 ng/mL), and Neural Serum Factor 1 (NSF1, 2%) (from the Lonza PNGM™ Singlequots™ Growth Supplements) and used for cell culture.

Following the sterilization of the chips occurred within the cell culture hood and by exposure to UV light for 30 minutes, the culture surface area of the MEAs was coated with a solution of poly-D-lysine (30 µg/mL, Sigma-Aldrich, St. Louis, MO, USA) and laminin (2 µg/mL, Sigma-Aldrich, St. Louis, MO, USA) in Phosphate Buffer Saline (PBS, pH = 7.4, Thermo Fisher Scientific, Inc., Waltham, MA) for 1 h at room temperature, to increase the cells adhesion on the chips. Thus, the MEAs were rinsed three times with sterile water (Sigma-Aldrich, St. Louis, MO, USA) and dried inside the hood, before cell seeding. Primary rat hippocampal neurons were seeded on the devices and incubated (37 °C, 5% CO_2, 95% humidity) for 4 h. Following adhesion, a portion of the medium was gently removed, and fresh and pre-warmed medium was introduced. The cell culture was sustained for 3 weeks. On day 5, half of the medium was replaced with fresh and pre-warmed medium. The same procedure was routinely carried out every 3-4 days until the spectroscopical and electrophysiological measurements.

Raman spectroscopy

A Renishaw InVia was used for Raman imaging. The samples were imaged using a 532 nm wavelength of excitation, laser power of 13 mW at the objective, a 60x water immersive lens objective (NA=1), peak center set at 1200 cm^{-1}, integration time of 0.4 seconds, 1 accumulation, step size acquisition of 3 µm and high confocality mode. The size of the Raman maps collected in the simultaneous electrophysiological-spectroscopic analysis was 30x30 µm^2. The required time for recording a 30x30 µm^2 Raman map is 2.5 minutes.

Data processing

Raman data were collected and processed in two stages, the preprocessing occurs using WiRE 5.5 and proceeds as follows: truncation, cosmic rays removal, noise filter, subtraction baseline and smooth (Figure S1, panel 2). The truncation was carried out maintaining the Raman spectrum from 500 to 1720 cm^{-1}. The cosmic rays removal was carried out in two steps, (i) using a detection method with width parameter and height parameter set as 3 and 15 and (ii) using the nearest neighbour algorithm with noise level set as 0.89 and scaling factor 10 as well as spectrum height set as 6.63 and scaling factor 50%. The noise filter was based upon a WiRE 5.5 algorithm of principal component analysis which results in the generation of a software interface in which the components (expressed as loadings) can be manually selected to discriminate the noise from the Raman signals. The subtraction of the baseline occurred using polynomial order and noise tolerance as 12 and 1.5, respectively. Finally, the Raman spectra were smoothed using the Savitski-Golay filter with smooth window and polynomial order set as 9 and 3, respectively.

Then, the pre-processed data set was imported in MATLAB where it was further processed. The MATLAB script used in this work was written implementing an existing one.[14,15] The pre-processed

data set was normalized to the Raman scattering intensity corresponding to the phenylalanine at about 1004 cm^{-1} and then used to calculate the median corresponding to each Raman map, prior to and following the gabazine exposure (Figure S1, panel 2). The accuracy in the calculation of the median Raman spectrum for each Raman map was therefore depending upon the step size acquisition and the size of the designed area. Then, the five medians per sample associated with each condition, prior to and following gabazine exposure (30 µM, 10 mins), were averaged to generate a representative Raman spectrum of the sample prior to and following the chemical treatment (Figure S1, panel 3). We carried out this procedure for nine independent samples over four different neuronal cultures (Figure S1, panel 4), resulting in the collection and analysis of a total of 10890 Raman spectra, 5445 for each condition. The whole data analysis pipeline schematic is shown in Figure S1.

Electrophysiological measurements

Electrophysiological measurements were carried out using a MEA2100 (MEA2100-LITE-System) utilizing TiN MEAs with an electrode diameter of 30 µm and a spacing of 200 µm. High pass and low pass filters were set at 100 Hz (2nd order) and 3500 Hz (4th order) while the sample rate was set at 25 kHz. The neuronal activity considering the whole active electrodes was calculated through the Multi Channel Analyzer and included the following variables: firing rate, burst frequency, burst interval, burst duration and percentage of spikes within bursts.

The analysis was carried out using the default spike detection and analysis parameters. Specifically, the detection of the single spikes was based upon the negative phase of the neuronal action potential. Spikes were identified either by applying a threshold set at five times the standard deviation of the background noise or by manually defining a threshold across all active electrodes. The manual thresholding approach was employed when spike amplitudes or noise levels varied significantly across electrodes, making a uniform threshold unreliable. In such cases, electrode-specific thresholds were applied, based on the individual signal-to-noise ratio of each channel. Importantly, the same threshold values were consistently maintained before and after gabazine exposure to ensure unbiased extraction of electrophysiological features. It should be noted that the accuracy of spike detection was primarily dependent on the initial thresholding method. The electrophysiological recording was carried out for circa 600 seconds prior to and following the gabazine (30 µM, 10 mins) or DMSO (0.1%, 10 mins) exposure. DMSO was used to dissolve gabazine and therefore has been used as vehicle control.

Data analysis

Single feature analysis was carried out using the non-parametric Mann-Whitney test. The significance level was set at 0.05. The cross-correlation analysis was carried out using the Pearson correlation app in OriginPro 2025 (correlation plot). The significance level was set at 0.05.

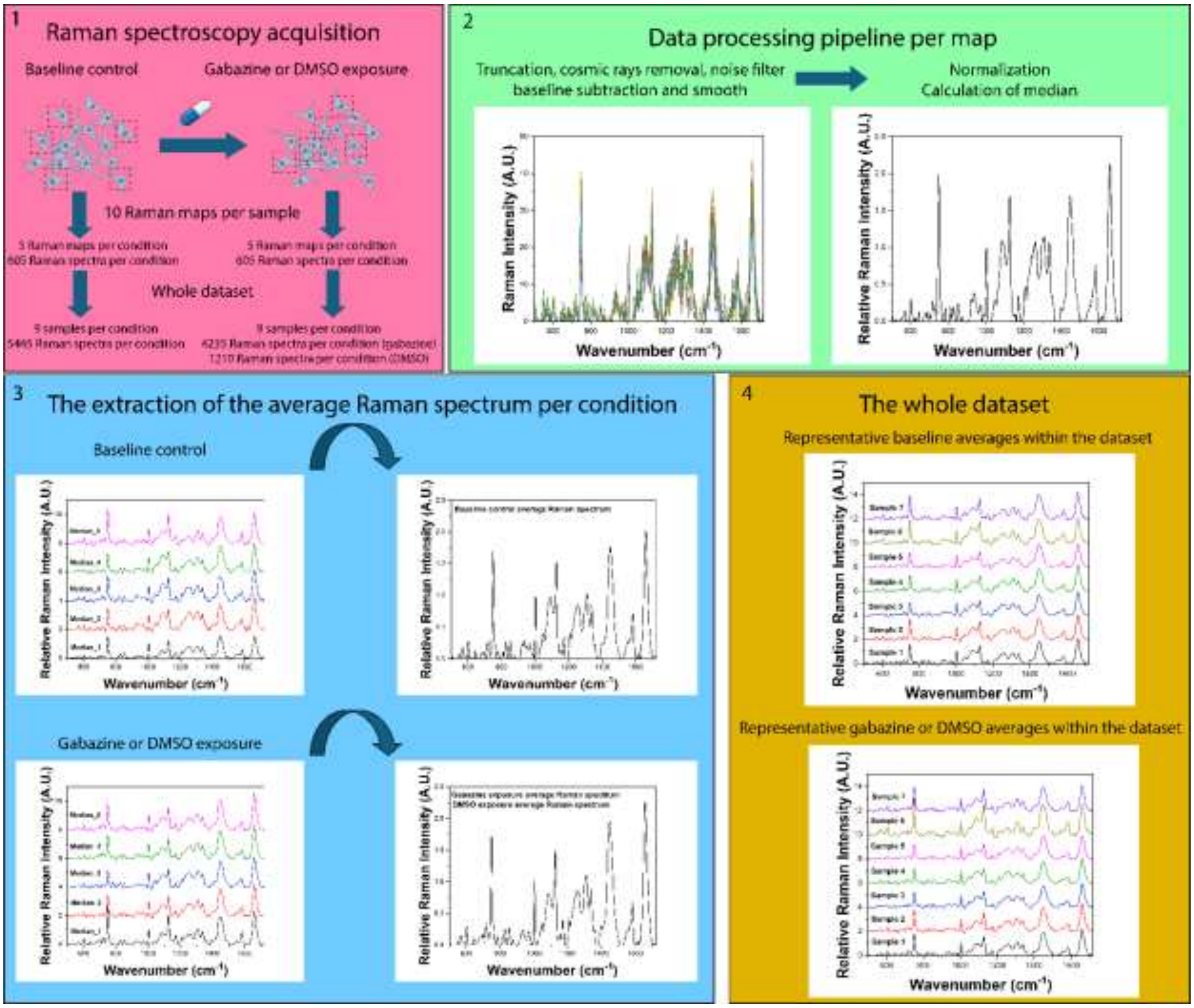

Figure S1. The data processing pipeline schematics used in this work for the spectroscopical data processing. Panel 1 shows an overview of the Raman measurements specifics, including the number of Raman maps collected per sample, the Raman maps collected per condition, the number of Raman spectra collected per condition and sample as well as the total number of Raman spectra collected per condition within the whole dataset. Panel 2 shows the data processing of the Raman spectra per each map, from the initial preprocessing in WiRE (truncate, cosmic rays removal, noise filter, baseline subtraction and smoothing) to the last processing steps in MATLAB (normalization and calculation of median per Raman map). Panel 3 shows the extraction of the average Raman spectrum per condition in each sample considering the five Raman maps collected per condition in each sample. Panel 4 shows the representative average Raman spectrum per condition for some of the samples analyzed in this work. A total of nine paired samples and four different neuronal cultures have been investigated in this work.

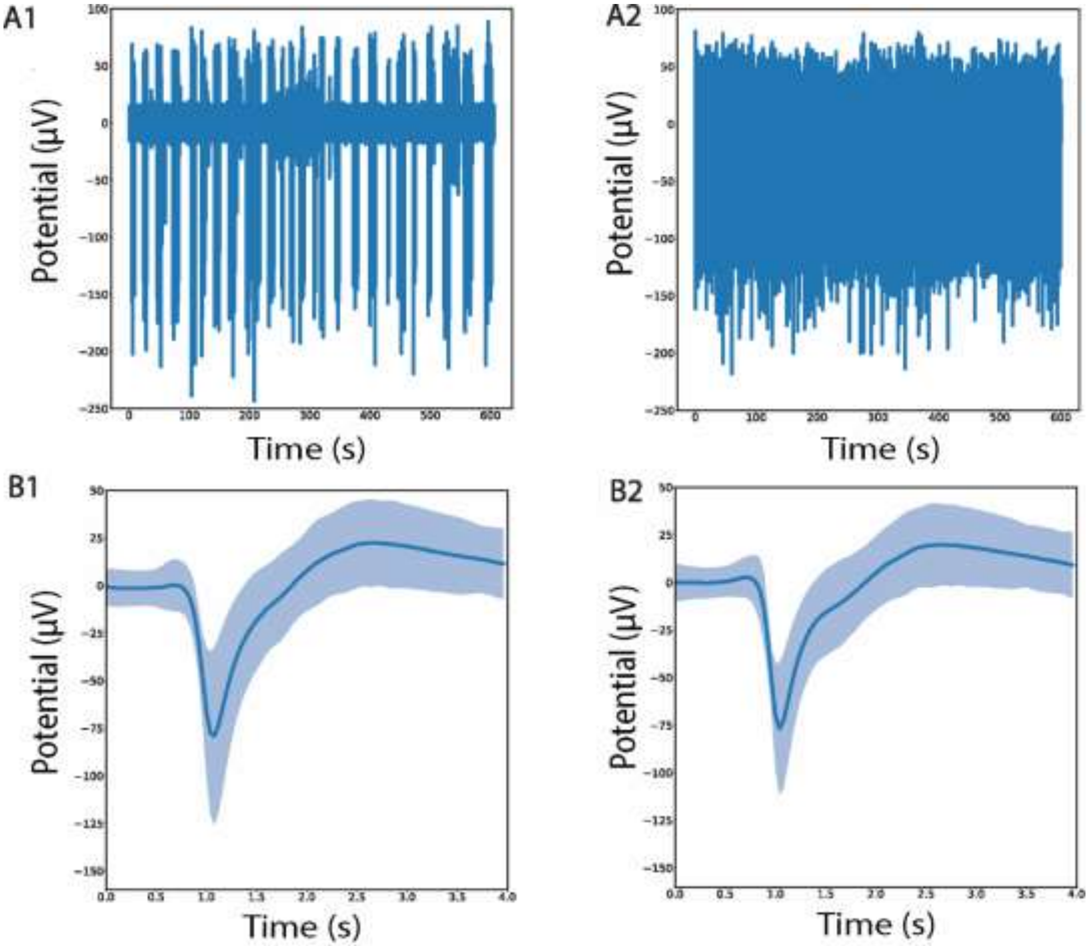

Figure S2. A representative electrophysiological activity of the primary neuronal rat cells, at an electrode of interest, prior to and following the gabazine exposure (30 μM,10 mins). The electrophysiological readout of the primary neuronal rat cells (A1) prior to and (A2) following the gabazine exposure as a plot of the potential changes at the electrode of interest over time. The average waveform and its standard deviation associated with the electrophysiological activity of neuronal primary rat cells (B1) prior to and (B2) following the gabazine exposure at an electrode of interest.

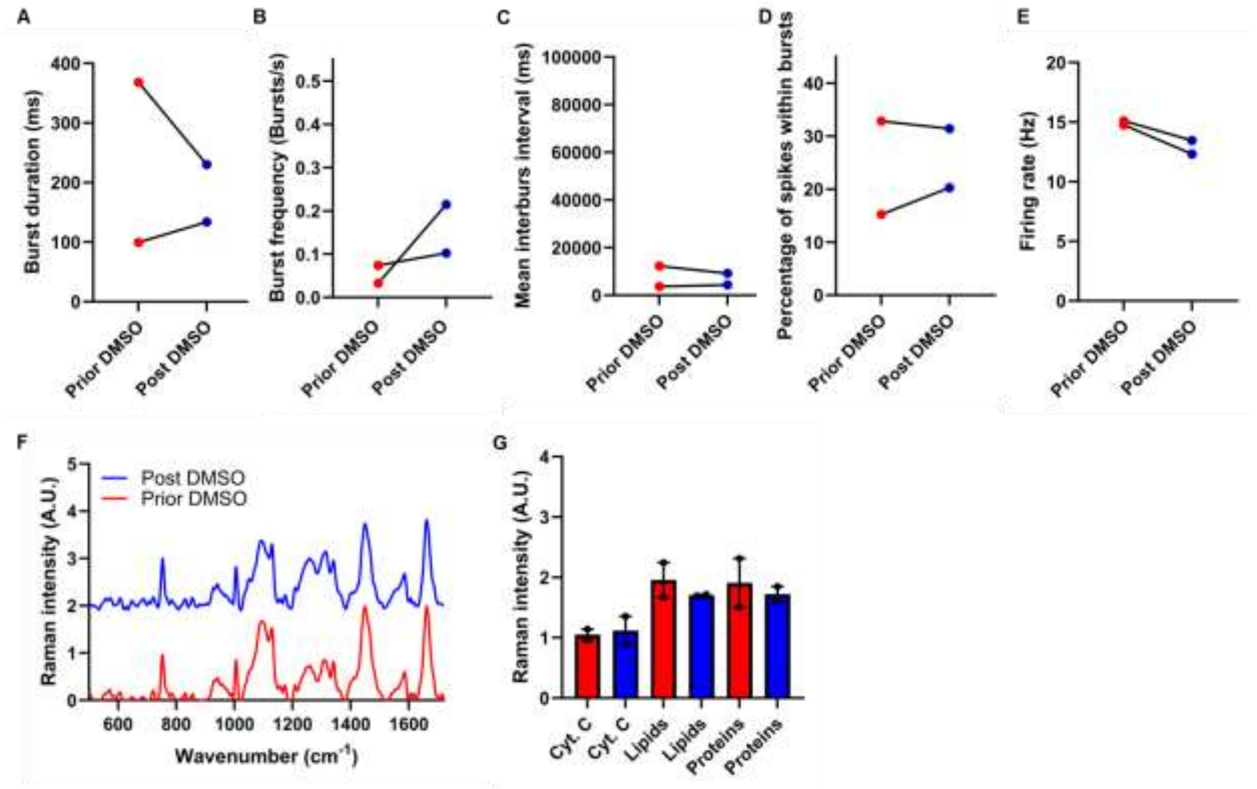

Figure S3. The electrophysiological and vibrational features of the neuronal cell culture prior to or following the treatment with the vehicle control (0.1% DMSO, 10 mins). (A) The changes across the burst duration in primary neuronal rat cells prior to and following the treatment with the vehicle control (0.1% DMSO, 10 mins). (B) The changes across the burst frequency in primary neuronal rat cells prior to and following the treatment with the vehicle control (0.1% DMSO, 10 mins). (C) The changes across the interburst intervals in primary neuronal rat cells prior to and following the treatment with the vehicle control (0.1% DMSO, 10 mins). (D) The changes across the percentage of spikes within bursts in primary neuronal rat cells prior to and following the treatment with the vehicle control (0.1% DMSO, 10 mins). (E) The changes in the firing rate of primary neuronal rat cells prior to and following the treatment with the vehicle control (0.1% DMSO, 10 mins). F) The average Raman spectrum of primary neuronal rat cells measured from two neuronal cell cultures prior to and following the treatment with the vehicle control (0.1% DMSO, 10 mins). (G) The relative Raman scattering of cytochrome C (750/1004) cm^{-1}, lipids (1450/1004) cm^{-1} and proteins (1660/1004) cm^{-1} prior to (red) and following the treatment with the vehicle control (0.1% DMSO, 10 mins) (blue). The data are illustrated as median (bar values) ± IQR. The single dots in each column represent the average relative intensity values calculated for two different neuronal cultures, and samples, prior to and following the treatment with the vehicle control (0.1% DMSO, 10 mins).

Baseline control	
**Feature**	**Single values**
Cytochrome C (750/1004) cm^{-1}	1.58; 1.69; 1.06; 1.35; 1.92; 1.88; 1.22
Lipids (1450/1004) cm^{-1}	1.83; 1.76; 1.74; 1.91; 2.03; 2.01; 1.92
Proteins (1660/1004) cm^{-1}	2.06; 2.02; 1.93; 2.16; 2.17; 2.04; 1.93
Burst frequency (burst/s)	0.05; 0.09; 0.02; 0.04; 0.17; 0.11; 0.01
Burst duration (ms)	24.46; 106.81; 49.74; 32.63; 279.58; 212.03; 83.90
Percentage of spikes within bursts	3.22; 7.16; 2.81; 3.54; 38.00; 38.03; 3.85
Firing rate (Hz)	3.48; 8.85 3.93; 2.77; 12.30; 4.73; 2.53
Interburst interval (ms)	17847.26; 6607.24; 36217.81; 23803.70; 5705.59; 7970.15; 80284.85

Upon gabazine exposure	
**Feature**	**Single values**
Cytochrome C (750/1004) cm^{-1}	1.71; 1.74; 1.23; 1.16; 1.91; 3.00; 1.20
Lipids (1450/1004) cm^{-1}	1.91; 1.95; 1.76; 1.75; 1.96; 2.44; 1.93
Proteins (1660/1004) cm^{-1}	2.20; 2.26; 1.95; 2.01; 2.12; 2.50; 1.94
Burst frequency (s^{-1})	0.08; 0.03; 0.44; 0.08; 0.09; 0.32; 0.005
Burst duration (ms)	14.81; 12.96; 3.21; 11.76; 206.30; 40.29; 101.20
Percentage of spikes within bursts	2.75; 0.56; 4.28; 2.66; 11.46; 26.53; 0.90
Firing rate (Hz)	5.57; 13.16; 17.14; 5.33; 14.66; 10.57; 3.51
Interburst interval (ms)	10992.81; 30348.78; 2224.66; 11524.86; 10749.74; 2979.41; 65067.68

Table S1. The singular values associated with each electrophysiological and spectroscopical feature, prior to and following the gabazine exposure (30 µM, 10 mins).

Feature	Mann-Whitney test
Cytochrome C	0.7983
Lipids	0.7983
Proteins	0.37109
Burst frequency	0.70148
Burst duration	0.0967
Percentage of spikes within bursts	0.20134
Firing rate	0.05528
Interburst interval	0.60928

Table S2. The Mann-Whitney test values associated with each dataset feature calculated from the comparison between the baseline control and the gabazine exposure (30 µM, 10 mins). The Mann-Whitney tests were carried out over seven independent samples and two different neuronal cultures.

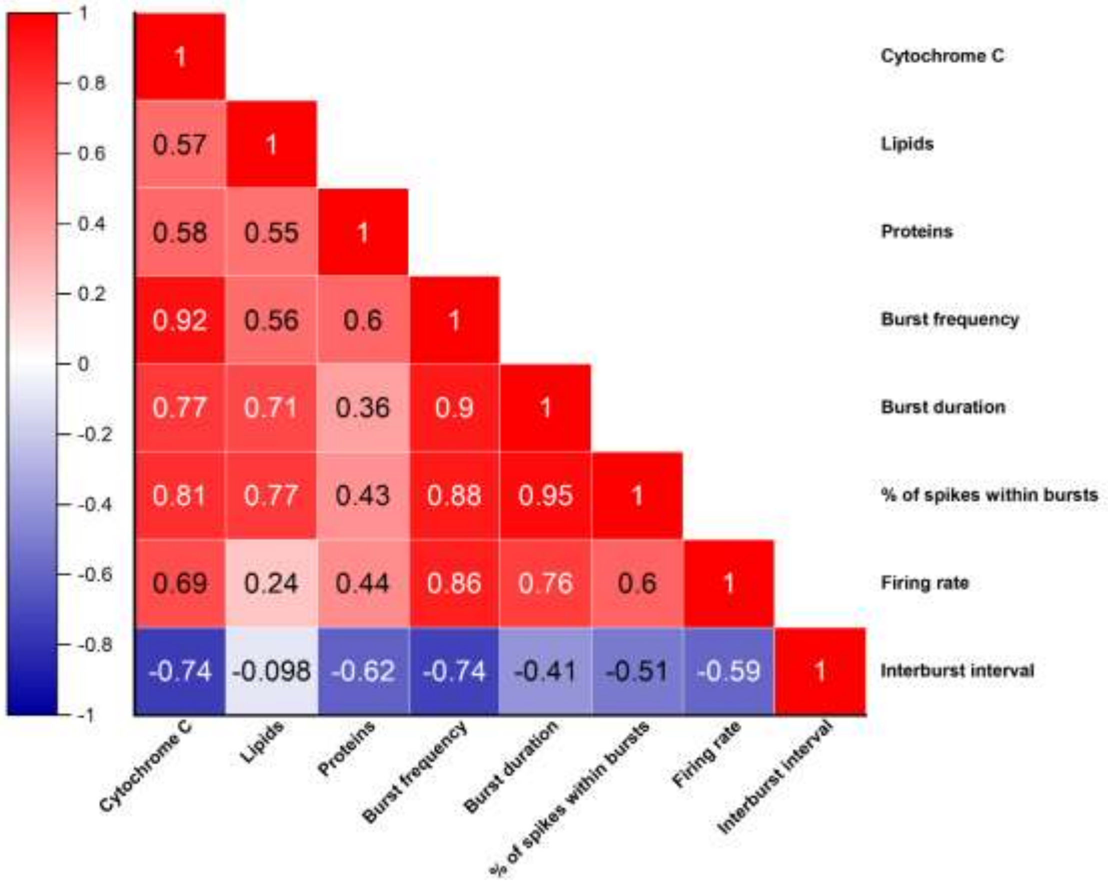

Figure S4. The Pearson correlation values among bio features prior to the gabazine exposure (30 μM, 10 mins). The Pearson correlation was carried out over seven independent samples and two different neuronal cultures.

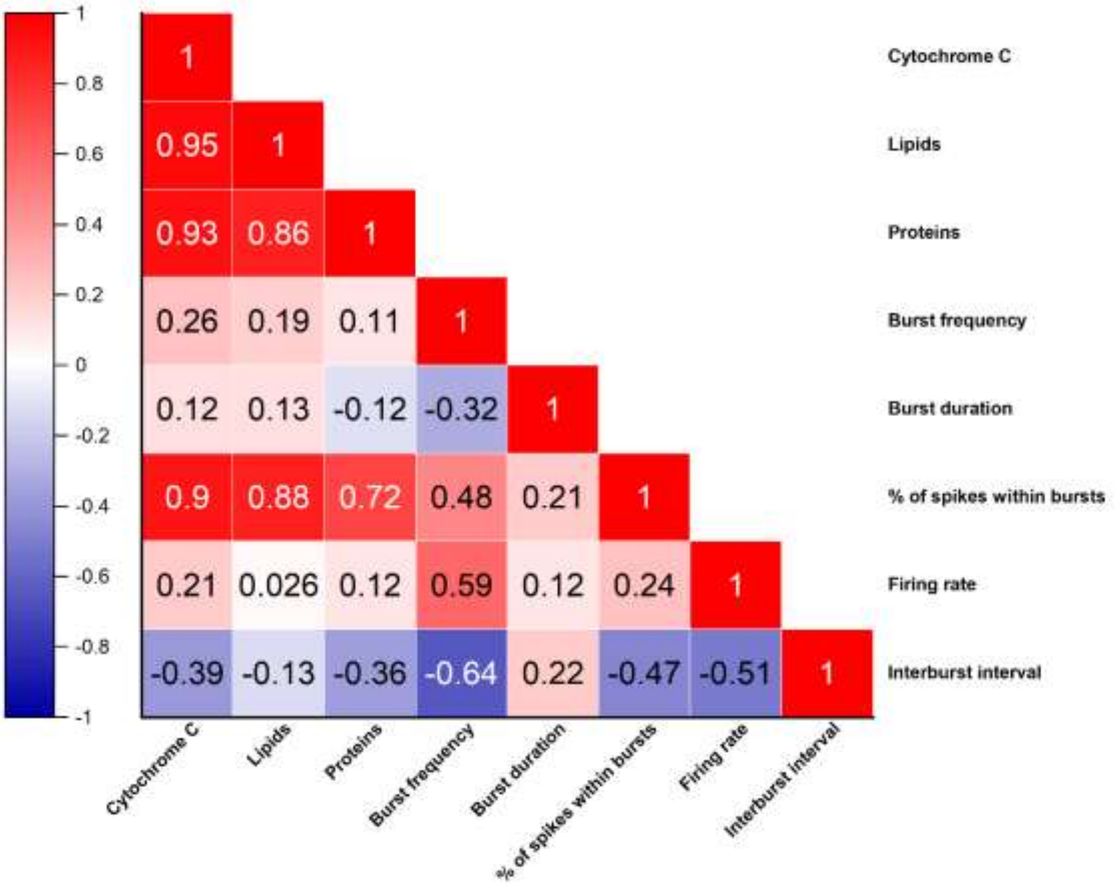

Figure S5. The Pearson correlation values among bio features following the gabazine exposure (30 µM, 10 mins). The Pearson correlation was carried out over seven independent samples and two different neuronal cultures.

References:

[1] Tentellino, C.; d'Amora, M.; Melikov, R.; Iachetta, G.; Bruno, G.; Tantussi, F.; Dipalo, M.; De Angelis, F. Electrode- and Label-Free Assessment of Electrophysiological Firing Rates through Cytochrome C Monitoring via Raman Spectroscopy. *ACS Sensors*, 2025, 10, 2, 1228-1236. DOI: 10.1021/acssensors.4c03133
